# Les leucémies aiguës lymphoblastiques de l’enfant à Ouagadougou (Burkina Faso): résultats de la prise en charge selon le protocole du Groupe Franco-Africain d’Oncologie Pédiatrique 2005

**DOI:** 10.11604/pamj.2018.29.44.11902

**Published:** 2018-01-18

**Authors:** Sonia Douamba, Fatimata Diallo, Kisito Nagalo, Laure Tamini, Lassina Dao, Fla Kouéta, Diarra Yé

**Affiliations:** 1 Service de la Pédiatrie Médicale du CHU Pédiatrique Charles de Gaulle de Ouagadougou, Burkina Faso; 2 UFR SDS Université Ouaga I Pr Joseph Ki-Zerbo de Ouagadougou, Burkina Faso

**Keywords:** Leucémie aiguë lymphoblastique, enfant, Groupe Franco-Africain d´Oncologie Pédiatrique, Acute lymphoblastic leukemia, child, Franco-African pediatric oncology group

## Abstract

**Introduction:**

La leucémie aiguë lymphoblastique (LAL) de l'enfant est une pathologie de plus en plus diagnostiquée dans notre service. Dans les pays développés, le traitement de cette hémopathie maligne permet de guérir près de 80% des enfants. Dans les pays en développement, peu d'études sont consacrées aux leucémies aiguës chez l'enfant. Les résultats du traitement des cancers de l'enfant sont décevants dans la plupart des pays africains avec un taux de survie de l'ordre de 10 à 15%. Le but de cette étude était d'étudier les aspects cliniques, biologiques, thérapeutiques et évolutifs des cas de LAL de l'enfant.

**Méthodes:**

Il s'agissait d'une étude rétrospective sur dossiers des enfants hospitalisés pour LAL entre Novembre 2009 et Octobre 2011 dans l'unité pilote d'oncologie pédiatrique du Centre Hospitalier Universitaire Pédiatrique Charles De Gaulle de Ouagadougou (Burkina Faso). Etaient inclus, les enfants pris en charge selon le protocole du Groupe Franco-Africain d'Oncologie Pédiatrique (GFAOP) 2005.

**Résultats:**

Au total, neuf cas de LAL étaient hospitalisés pendant les deux années étudiées. L'âge moyen des patients était 10,77 ans ± 2,82 ans. On notait une prédominance masculine. Le délai moyen d'hospitalisation était 43,11 jours ± 39,54 jours. Les principaux signes d'appel étaient l'altération de l'état général et la fièvre. Le syndrome tumoral et d'insuffisance médullaire étaient présents chez la quasi-totalité des patients. Six des neuf patients présentaient une LAL de type 1 au myélogramme. Huit patients ont bénéficié de la chimiothérapie selon le protocole du GFAOP 2005. L'évolution était favorable chez deux patients avec une rémission, quatre patients étaient en échec de traitement. Six patients sont décédés.

**Conclusion:**

Grâce à des campagnes d'information qui contribueront à amener la population à consulter précocement, au renforcement des capacités du personnel qui permettra un diagnostic précoce des LAL, la construction d'un centre d'oncologie pédiatrique suffisamment équipé et une subvention par l'Etat burkinabè des médicaments anticancéreux, la prise en charge de la LAL chez l'enfant permettraient d'obtenir de meilleurs résultats.

## Abstract (en)

**Introduction:**

acute lymphoblastic leukemia (ALL) is being diagnosed in an increasing number of children in our Department. In the developed countries, the treatment of this hematologic malignancy can cure almost 80% of children. In developing countries, few studies focus on acute leukemias in children. The results of cancer treatments in children are disappointing in most African countries, with a survival rate of 10-15%. This study aimed to investigate the clinical, biological, therapeutic and evolutionary features of ALL in children.

**Methods:**

we conducted a retrospective study of the medical records of children hospitalized for ALL between November 2009 and October 2011 in the pilot Paediatric Oncology Unit at the Charles de Gaulle University Pediatric Hospital Center, Ouagadougou (Burkina Faso). All children treated according to the protocol of the Franco-African Pediatric Oncology Group 2005 (FAPOG) were included in the study.

**Results:**

in total, nine children with ALL were hospitalized during the two year study period. The average age of patients was 10.77± 2.82 years. They were predominantly male. The average time of hospitalization was 43.11 days ± 39.54 days. The main symptoms were alteration of general state and fever. Nearly all the patients had tumor syndrome and bone marrow failure. Myelogram showed ALL type 1 in six of the nine patients. Eight patients underwent chemotherapy according the protocol of FAPOG 2005. Children’s evolution was favorable in two patients who experienced remission, four patients had treatment failure. Six patients died.

**Conclusion:**

thanks to information campaigns, which will contribute to encourage early consultations, capacity-building measures for the medical staff allowing early diagnosis of ALL, the construction of a sufficiently equipped pediatric oncology center and a subsidy of anticancer drugs awarded by the state of Burkina Faso, the treatment of children with ALL would allow for better outcomes.

## Introduction

Les leucémies aiguës lymphoblastiques (LAL) sont des proliférations malignes monoclonales envahissant la moelle osseuse, développées à partir de cellules lymphoïdes bloquées à un stade précoce de la différenciation cellulaire et incapables de maturation terminale. Il en résulte la survenue d'un tableau clinico-biologique d'insuffisance médullaire [1]. Les leucémies constituent le cancer le plus fréquent chez l'enfant, jusqu'à 30% des cancers de l'enfant dans les pays occidentaux et environ 400 nouveaux cas sont diagnostiqués chaque année en France. Dans les pays développés, le traitement des LAL de l'enfant permet actuellement de guérir près de 80% des enfants [2]. Dans les pays en développement, il existe peu d'études sur les cancers de l'enfant et particulièrement sur les leucémies aiguës. Cela est dû à l'ampleur des maladies infectieuses et nutritionnelles qui sont plus préoccupantes. Néanmoins certaines études placent les leucémies et les lymphomes au premier rang des cancers chez l'enfant de moins de 15 ans [3]. Au Burkina Faso, sur une décennie, 25 cas de LA étaient répertoriés dans la ville de Ouagadougou [4]. Dans la plupart des pays africains, les résultats du traitement des cancers de l'enfant sont décevants en montrant un taux de survie de 10 à 15% seulement [5]. Le Groupe Franco-Africain d'Oncologie Pédiatrique (GFAOP) s'est constitué en Octobre 2000 et s'est fixé pour objectif l'améliorer cette situation défavorable en Afrique. Les activités du GFAOP au Burkina Faso ont débuté en 2005 avec la prise en charge des lymphomes, des néphroblastomes et plus récemment celle des leucémies aiguës lymphoblastiques depuis Novembre 2009. Notre étude avait pour but d'étudier les aspects clinico-biologiques, et évolutifs des LAL de l'enfant traité au CHUP-CDG selon le protocole du GFAOP 2005.

## Méthodes

**Cadre de l'étude:** L'étude s'est déroulée dans le service de pédiatrie médicale du CHUP/CDG, au sein de l'Unité des grands enfants. Dans cette Unité dont la capacité est de 30 lits, huit lits sont réservés à l'Oncologie.

**Méthode:** Il s'agissait d'une étude rétrospective avec revue des dossiers des cas de LAL de l'enfant pris en charge selon le protocole du GFAOP LAL 2005 dans l'unité d'oncologie du service de pédiatrie médicale, dans la période allant du 1^er^ Novembre 2009 au 31 Octobre 2011. Etait inclus dans cette étude, tout enfant âgé de 1 à 15 ans hospitalisé pour LAL traité selon le protocole GFAOP, tout cas de LAL en 1^ère^ poussée, toute cytologie de la classification Franco-Américano-Britannique (FAB) L1 ou L2, leucocytose < 100 000 globules blancs/mm^3^ pour les enfants dont l'âge est compris entre 1 et 10 ans, leucocytose < 50 000 globules blancs/mm^3^ pour les enfants dont l'âge ≥ 10 ans, absence d'élargissement du médiastin, absence de chimiothérapie préalable.

**Aspects éthiques de l'étude:** L'anonymat et la confidentialité des données ont été respectés au cours de cette étude.

## Résultats

### Aspects épidémiologiques

***Fréquence:*** Durant les deux années couvertes par l'étude, 39 enfants étaient hospitalisés dans l'Unité d'oncologie. Parmi ceux-ci, neuf avaient une LAL, soit une fréquence de 23,08%.

***Sexe et âge:*** Sur les neuf enfants atteints de LAL, quatre étaient de sexe féminin et cinq de sexe masculin (sex-ratio de 1,25). L'âge moyen des enfants était 10,77 ans ± 2,82 ans (7-15 ans).

**Aspects cliniques:** Un patient sur les neuf atteints de LAL avait un antécédent d'épistaxis. Il n'y avait a pas d'antécédent familial de LAL. Les principaux motifs de consultation étaient la fièvre et les douleurs osseuses (six cas sur les neuf). Le délai moyen d'hospitalisation était de 43,11 jours ± 39,54 jours (3-120 jours). L'état général était altéré chez sept patients et conservé chez deux patients. Le syndrome tumoral était associé au syndrome d'insuffisance médullaire dans huit cas.

**Aspects paracliniques:** A l'hémogramme, le taux moyen de leucocytes était de 73244,44 ± 100642,11/mm^3^(1700-263400/mm^3^). Six patients avaient une hyperleucocytose. Le taux moyen de lymphocytes était de 54,87% ± 31,31% (3-98%). Une neutropénie sévère (polynucléaires neutrophiles (< 500/mm^3^) était rencontrée chez trois patients. L'anémie était présente chez tous les patients; le taux moyen d'hémoglobine moyen était 6,93 ± 1,47 g/dl (5,1-9,9 g/dl). La valeur moyenne des plaquettes était de 131777 ± 183859/mm^3^ (23000-608000/mm^3^). Sept patients avaient une thrombopénie. Au myélogramme, le taux moyen de lymphoblastes était de 72,55% (1-90%). Un patient sous corticothérapie au long cours avait un pourcentage de lymphoblastes à 10%. Six patients avaient une LAL de type 1, trois patients une LAL de type 2. La cytochimie, l'immunophénotypage et le caryotype n'ont pas être réalisés.

**Aspects thérapeutiques:** Huit patients ont été traités selon le protocole GFAOP 2005. Le délai moyen de la chimiothérapie était de 16,62 ± 12,95 jours (3-38 jours). L'aplasie était notée chez tous les huit patients et elle était associée à la fièvre chez cinq 5 patients à la période d'induction. A l'induction, la durée moyenne de l'aplasie était 30,62 jours. La durée moyenne de report de la chimiothérapie à cause de l'aplasie était 33,43 jours. Une rémission était obtenue chez deux patients. Elle était complète dans un cas et incomplète dans un autre cas (avec 7% de blastes au myélogramme). Chez deux patients le myélogramme de rémission n'a pas été réalisé. Trois patients ont présenté une rechute. Celle-ci était survenue à la période de consolidation (présence de blaste à 8% au frottis sanguin) chez un patient et à la période d'intensification chez les deux autres (réapparition du syndrome tumoral et du syndrome d'insuffisance médullaire). Quatre patients étaient en échec de traitement.

**Complications:** Les complications infectieuses étaient plus fréquentes (six patients) et dominées par la septicémie qui survenait surtout à la période d'induction. On notait aussi quatre cas d'hémorragies.

**Evolution:** Sur les neuf patients, deux étaient toujours vivants dont un en traitement d'entretien et suivi en ambulatoire, l'autre avait été évacué hors du pays où il poursuivait sa chimiothérapie. Sept patients étaient décédés dont un en phase de préparation, quatre en période d'induction et deux en période d'intensification. Les causes de décès étaient la septicémie dans six cas et l'évolution leucémique dans un cas. La durée moyenne d'hospitalisation était de 137,67 jours soit 4,59 mois (35-333 jours) ou (1,16-11,1 mois).

## Discussion

### Des aspects épidémiologiques

La fréquence hospitalière du LAL de l'enfant dans notre étude est superposable à celle des autres études africaines. En effet, en une décennie, Sawadogo et *al*. [3] et Ouédraogo et al. [4] rapportent respectivement 71 cas en Côte d'Ivoire et 20 cas au Burkina Faso. Togo et *al*. [6] rapportent quant à eux 12 cas de LAL en deux ans et demi au Mali tandis que Ka et al. [7] colligeaient 11 cas en 11 années au Sénégal. Cependant, cette fréquence dans les pays africains reste plus élevée que celle rapportée dans les pays développés [8]. Cette différence s'expliquerait en partie par l'existence d'une couverture sociale gage d'un meilleur diagnostic et d'un système de surveillance épidémiologique plus efficace dans ces pays développés. L'âge moyen des patients dans notre étude était légèrement plus élevé que celui rapporté en Afrique par Ouédraogo et *al*. [4], Ka [7] et Togo [6] qui trouvaient respectivement 6,82 ans, 7,16 ans et 7,66 ans. La différence observée est probablement liée au mode de recrutement des patients. Selon les données de la littérature, le pic de fréquence des LAL se situe entre 2 et 5 ans [9,10]. Nous avons noté une prédominance masculine dans notre étude, en concordance avec le constat fait par d'autres auteurs [4,7,11]. La surmorbidité masculine classique constatée en pédiatrie pourrait expliquer cette prédominance.

**Des aspects cliniques** Dans notre série, le délai moyen d'hospitalisation était d'un mois et demi. Ce long délai est observé dans les séries africaines avec des moyennes variables: 2,88 mois pour Ouédraogo et *al*. [4], trois mois pour Ka et *al*. [7]. Dans l'étude de Togo et *al*. [6], plus du tiers des patients avaient consulté cinq mois après le début de la maladie. Contrairement aux pays développés où le délai d'hospitalisation est plus bref, il y a un long délai d'hospitalisation des LAL dans les pays africains et cela expliquerait en partie le mauvais pronostic observé dans les études africaines. Dans notre étude, nous avons noté que l'altération de l'état général et la fièvre étaient les principaux signes d'appel du LAL. Notre résultat était similaire à celui d'autres auteurs d'Afrique subsaharienne [6,7]. L'altération fébrile de l'état général dans les LAL traduit souvent le retard à la consultation et réduirait les chances de guérison car les enfants sont amenés en consultation à un stade déjà avancé de la maladie, ce qui rend difficile la prise en charge. Le syndrome tumoral et le syndrome d'insuffisance médullaire étaient présents chez la quasi-totalité de nos patients. Ces deux syndromes rapportés par la plupart des auteurs [1,2,4,6] restent les maîtres symptômes permettant le diagnostic clinique du LAL.

### Des aspects paracliniques

Au myélogramme, selon le type cytologique, nous avons noté six cas de LAL 1 et trois cas de LAL 2. Nos résultats sont similaires à ceux d'autres auteurs africains qui rapportent aussi une plus grande fréquence de ces deux types de LAL [3,7]. Dans notre étude, la cytochimie, l'immunophénotypage, la cytogénétique et la biologie moléculaire n'étaient pas faits, faute de moyens financiers et d'un laboratoire local pouvant réaliser ces examens. Cette difficulté diagnostique est également partagée par d'autres auteurs de pays à ressources limitées [3,4,7].

### Des aspects thérapeutiques et évolutifs

Le protocole utilisé était le GFAOP LAL-2005, comme dans l'étude de Togo et *al*. [6]. Nos résultats, en termes de rémission, de complications et d'échecs thérapeutiques, et de décès, sont proches de ceux de Ka et *al*. [7] qui rapportent une seule guérison contre sept décès sur les huit patients dans leur étude. La survie globale était meilleure dans la série de Togo et *al*. [6] avec 66,7% après deux ans. Le traitement des affections malignes de l'enfant reste décevant dans les pays en développement, notamment en Afrique sub-saharienne avec un taux de guérison de l'ordre de 10 à 15% [1,5], alors qu'en Occident les progrès thérapeutiques permettent une guérison dans 75 à 80% des cancers pédiatriques tous types confondus. De nombreux obstacles expliquent les mauvais résultats observés dans les pays à ressources limitées: le retard à la consultation, les difficultés diagnostiques, le coût financier élevé du traitement, la non disponibilité/ou rupture des médicaments anticancéreux, la difficulté de prise en charge des complications intercurrentes, l'absence de cadre adapté à la prise en charge, l'absence de service de radiothérapie fonctionnel, la lassitude des familles devant la longueur et les complications des traitements [4,11]. Les mauvais résultats de notre série bien que limitée viennent confirmer les données de la littérature africaine.

## Conclusion

La prise en charge des leucémies aiguës lymphoblastiques de l'enfant dans notre hôpital est récente, beaucoup d'efforts restent à fournir afin d'obtenir de meilleurs résultats. Le mauvais pronostic est lié d'une part au diagnostic tardif et d'autre part à l'insuffisance de la prise en charge. Les mauvais résultats demandent l'implication des pouvoirs publics, des ONG et associations de lutte contre les cancers pour accompagner les activités du GFAOP dans le cadre d'une dynamisation de l'unité d'oncologie pédiatrique.

### Etat des connaissances actuelle sur le sujet

- Les leucémies et les lymphomes sont les cancers les plus fréquents de l'enfant;
- La prise en charge des leucémies de l'enfant au Burkina a débuté en 2009 (il y a donc huit années seulement);
- On sait donc peu de choses sur l'évolution et le pronostic sous traitement, en particulier avec le protocole du GFAOP.

### Contribution de notre étude à la connaissance

- le LAL de l'enfant est un cancer de mauvais pronostic au Burkina Faso;
- les obstacles à une bonne prise en charge sont identifiés.

## Conflits d’intérêts

Les auteurs ne déclarent aucun conflit d'intérêts.
